# The metastasis suppressor, NDRG1, inhibits “stemness” of colorectal cancer *via* down-regulation of nuclear β-catenin and CD44

**DOI:** 10.18632/oncotarget.5294

**Published:** 2015-09-18

**Authors:** Xiongzhi Wangpu, Xiao Yang, Jingkun Zhao, Jiaoyang Lu, Shaopei Guan, Jun Lu, Zaklina Kovacevic, Wensheng Liu, Lan Mi, Runsen Jin, Jing Sun, Fei Yue, Junjun Ma, Aiguo Lu, Des R. Richardson, Lishun Wang, Minhua Zheng

**Affiliations:** ^1^ Department of General Surgery, Ruijin Hospital, Shanghai Jiao Tong University School of Medicine, Shanghai, 200025, China; ^2^ Shanghai Minimally Invasive Surgery Center, Shanghai, 200025, China; ^3^ Molecular Pharmacology and Pathology Program, Department of Pathology and Bosch Institute, University of Sydney, Sydney, New South Wales, 2006, Australia; ^4^ Shanghai Institute of Digestive Surgery, Ruijin Hospital, Shanghai Jiao Tong University School of Medicine, Shanghai, 200025, China; ^5^ The Division of Translational Medicine, Minhang Hospital, Fudan University, Shanghai, 201199, China

**Keywords:** β-catenin, colorectal cancer, NDRG1, stem cell-like property, tumorigenesis

## Abstract

N-myc downstream-regulated gene 1 (NDRG1), has been identified as an important metastasis suppressor for colorectal cancer (CRC). In this study, we investigated: (1) the effects of NDRG1 on CRC stemness and tumorigenesis; (2) the molecular mechanisms involved; and (3) the relationship between NDRG1 expression and colorectal cancer prognosis. Our investigation demonstrated that CRC cells with silenced NDRG1 showed more tumorigenic ability and stem cell-like properties, such as: colony and sphere formation, chemoresistance, cell invasion, high expression of CD44, and tumorigenicity *in vivo*. Moreover, NDRG1 silencing reduced β-catenin expression on the cell membrane, while increasing its nuclear expression. The anti-tumor activity of NDRG1 was demonstrated to be mediated by preventing β-catenin nuclear translocation, as silencing of this latter molecule could reverse the effects of silencing NDRG1 expression. NDRG1 expression was also demonstrated to be negatively correlated to CRC prognosis. In addition, there was a negative correlation between NDRG1 and nuclear β-catenin and also NDRG1 and CD44 expression in clinical CRC specimens. Taken together, our investigation demonstrates that the anti-metastatic activity of NDRG1 in CRC occurs through the down-regulation of nuclear β-catenin and suggests that NDRG1 is a significant therapeutic target.

## INTRODUCTION

Colorectal cancer (CRC) is the third most common cancer and the fourth most prevalent cause of tumor-related death worldwide [1, 2]. Patients with advanced CRC have a poor prognosis due to the high rate of resistance to radiotherapy or chemotherapy, which leads to recurrence, metastasis and death [3, 4]. Although combined therapy for CRC has made great progress, there is still an urgent need to better understand CRC recurrence and metastasis in order to design more effective therapies [5].

The epithelial to mesenchymal transition (EMT) is an embryonic process during which polarized epithelial cells transform into a more motile and mesenchymal phenotype [6]. This reversible process generates obvious changes in cell morphology and tumor behavior, such as metastasis and chemoresistance [7, 8]. The crucial molecular events during the EMT include the loss of E-cadherin on the cell membrane and the up-regulation of transcription factors, such as Slug, Snail, Twist and ZEB1, *etc*. It is commonly regarded that the gain of a mesenchymal phenotype is associated with some functional traits of cancer stem cell-like cells (CSCs) [9, 10]. In addition, these CSC properties are considered to be closely correlated with tumor initiation, progression and metastasis, as well as resistance to chemotherapy [11–13]. However, some studies have demonstrated that although the EMT is essential for tumor metastasis, it is not a requirement for the acquisition of CSC properties [14, 15].

N-myc downstream-regulated gene 1 (NDRG1) is a potent metastasis suppressor in multiple tumor-types [16, 17]. The molecular mechanism of action of this protein involves its suppressive effects on a variety of tumorigenic signaling pathways [18–23]. Increasing evidence suggests that over-expression of NDRG1 is able to inhibit the invasion and metastasis of CRC and has a negative correlation with colorectal cancer prognosis [24, 25]. However, in contrast, some studies have demonstrated that NDRG1 promotes cell migration and invasion in hepatocellular carcinoma [26, 27]. Hence, the specific functions of NDRG1 in CRC remain to be further investigated. Our previous studies have shown that NDRG1 inhibits CRC migration and invasion through inhibiting the EMT and β-catenin nuclear translocation [18, 22]. Considering the close relationship between tumorigenesis and CSC properties, in the current study, we examined the effect of NDRG1 on tumorigenic potential and CSC traits of two CRC cell lines. In addition, we examined whether the variation of these neoplastic characteristics can be influenced by NDRG1-regulated β-catenin expression.

Herein, we demonstrate that low NDRG1 expression promotes CSC traits of CRC *in vitro* and tumorigenesis *in vivo* through up-regulation of nuclear β-catenin and CD44 expression, while these phenotypes can be reversed by down-regulation of β-catenin. Importantly, NDRG1 positive CRC cases were found to have a better prognosis than NDRG1 negative cases. Finally, there was a close negative correlation between NDRG1 and nuclear β-catenin and also NDRG1 and CD44 expression in the clinical CRC specimens. These findings demonstrate that the effect of NDRG1 on inhibiting nuclear β-catenin translocation and also CD44 expression plays an important role in preventing CRC progression.

## RESULTS

### NDRG1 inhibits CSC-related phenotypes and tumorigenesis *in vitro*

Sphere formation is considered as one important characteristic of CSCs *in vitro* [28, 29]. These CSCs have strong tumorigenic potential, including the ability to metastasize, form colonies and display resistance to cytotoxic drugs, *etc.* [30, 31]. To examine the relationship between NDRG1 and these CSC-related properties, we performed a number of assays to assess sphere formation, metastasis, soft-agar colony formation and chemoresistance. These assays were performed using CRC cells, namely the HT29 and HCT116 cell lines, which were stably transfected to either over-express NDRG1 (labeled “NDRG1”) or silence NDRG1 (labeled as “sh NDRG1”), as previously used in our laboratories [21]. These cell lines are compared to the relevant controls transfected with the empty vector, namely: “NDRG1 Con” and “sh Con”, respectively.

**Figure 1:**
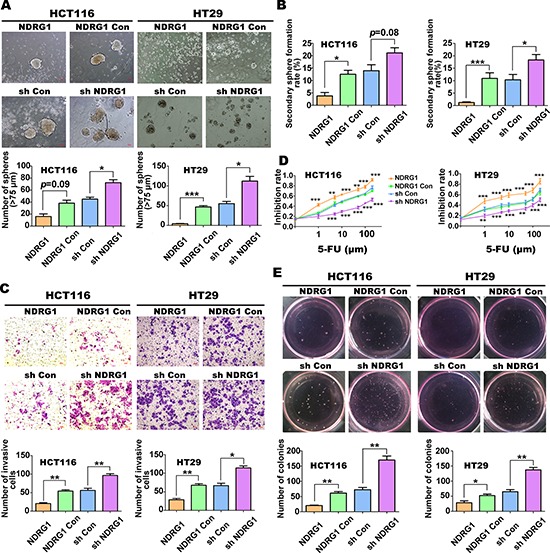
NDRG1 inhibits CSC-related phenotypes and tumorigenesis in CRC cells (HCT116 or HT29) with NDRG1 over-expression or silencing **A.** Comparison of sphere formation between HCT116 or HT29 cell-types with either NDRG1 over-expression (*i.e*., NDRG1 *vs*. “NDRG1 Con” [vector control]) or NDRG1 silencing (*i.e*., “sh Con” [vector control] *vs*. “sh NDRG1” [NDRG1 silenced]). **B.** Spheres were digested into single cells and secondary sphere formation rates were calculated to demonstrate different self-renewal ability. **C.** Transwell chambers were used to evaluate invasive ability of different NDRG1 expressing HCT116 and HT29 cells. **D.** Cell count (Cell Counting Kit8; CCK8) assays were performed after incubating NDRG1 over-expressing HCT116 and HT29 cells or their NDRG1 silenced counterparts with various concentrations of 5-FU. *p* values were calculated at respective concentrations. **E.** Effect of NDRG1 expression on colony formation ability in HCT116 and HT29 cells. All data are shown as mean ± SD (*n* = 3–6). *p < 0.05; **p < 0.01; ***p < 0.001.

Examining primary sphere formation of these cell lines (Fig. 1A), it was demonstrated that the number of spheres (diameter ≥ 75 μm) was reduced (*p* = 0.09) in HCT116 cells over-expressing NDRG1 when compared to its control group (NDRG1 Con). This effect of NDRG1 over-expression on inhibiting primary sphere formation was more pronounced in HT29 cells, where there was a marked and significant (*p* < 0.001) decrease relative to the NDRG1 Con (Fig. 1A). Furthermore, in both sh NDRG1 HCT116 and HT29 cells, spheroid formation was significantly (*p* < 0.05) increased relative to the sh Con cells (Fig. 1A). A similar trend in terms of the effect of NDRG1 expression was also observed upon re-suspension of the spheres and assessing secondary sphere formation (Fig. 1B). Collectively, these observations indicated that over-expression or silencing of NDRG1 either inhibited or enhanced, respectively, the renewal ability of sphere-derived CRC cells.

Utilizing a cell invasion assay (Fig. 1C), NDRG1 over-expression was shown to significantly (*p* < 0.01) result in lower rates of HCT116 and HT29 cell invasion when compared to the NDRG1 Con cells (Fig. 1C). Conversely, sh NDRG1 HCT116 and HT29 cells had significantly (*p* < 0.01–0.05) greater rates of invasion compared to their relevant sh Con cells (Fig. 1C). These results demonstrate that NDRG1 over-expression or silencing inhibits or enhances, respectively, the invasive potential of CRC cells, in agreement with our previous findings [18, 21].

Examining chemoresistance, we found that there were no significant differences (less than 20%) between the cell lines examined when they were incubated with a low concentration of the cytotoxic agent 5-fluorouracil (5-FU; 0.1 μM; data not shown). However, increasing the concentration of 5-FU from 1 to 100 μM, revealed that both the HT29 and HCT116 cells over-expressing NDRG1 were significantly (*p* < 0.001–0.01) more sensitive to this agent relative to the NDRG1 Con (Fig. 1D). Conversely, NDRG1 silencing in both cell-types significantly (*p* < 0.001–0.01) decreased the sensitivity to 5-FU at concentrations of 1 μM or higher relative to the sh Con (Fig. 1D).

Finally, upon examining colony formation using both HCT116 and HT29 cells, these studies demonstrated that NDRG1 over-expression resulted in a significant (*p* < 0.01–0.05) decrease in colony number, there being approximately half as many colonies as when compared to NDRG1 Con cells (Fig. 1E). In contrast, assessment of colony formation in sh NDRG1 cells from both cell-types demonstrated that there was a significant (*p* < 0.01) increase in colony formation (approximately 2-fold) relative to the sh Con cells (Fig. 1E). Collectively, these results in Figure 1 provide further evidence that NDRG1 expression inhibits CSC traits and tumorigenesis of CRC cells *in vitro*.

### NDRG1 down-regulates the expression of CSC surface marker, CD44, while having no effect on CD133

**Figure 2:**
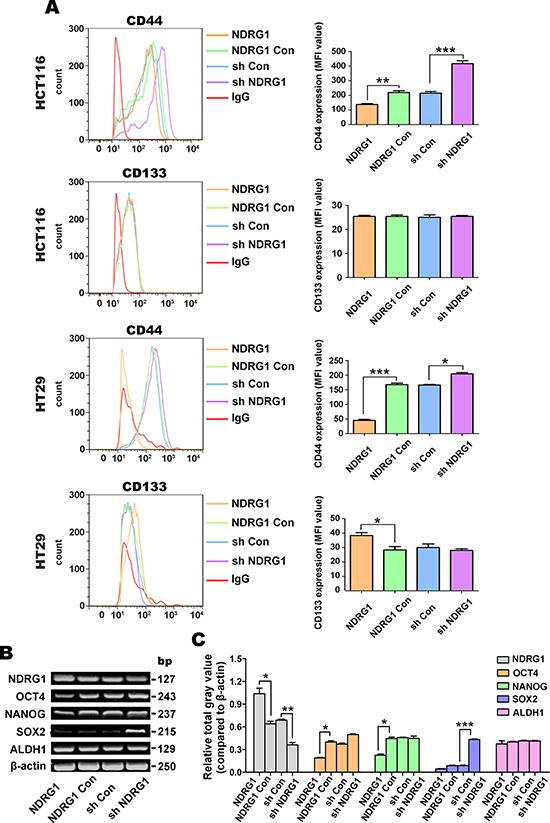
NDRG1 over-expression decreases expression of the CSC marker, CD44, but not CD133, in the HCT116 and HT29 cell-types **A.** Detection of the CSC surface markers, CD44 and CD133, using flow cytometry. PE-labeled mouse IgG2a was used as the isotype control. **B.** Reverse transcriptase PCR (RT-PCR) was implemented to investigate the expression of stem cell markers, including: *OCT4*, *NANOG*, *SOX2* and *ALDH1* mRNA. β-actin was used as the loading control. **C.** Densitometry of the RT-PCR results (*i.e*., relative total gray values standardized to β-actin were calculated). All data are shown as mean ± SD (*n* = 3). *p < 0.05; **p < 0.01; ***p < 0.001.

An increasing number of different CSC markers have recently been identified, although CD44 and CD133 are regarded as classical surface markers to screen CSCs from CRC cells [32–34]. Considering that NDRG1 may function as a negative regulator of CRC stemness, we investigated whether NDRG1 influences the expression of CD44 and CD133 by flow cytometry in both cell-types (Fig. 2A), as both these markers are cell surface-associated [35, 36]. To further support these results, we then examined the mRNA expression of other well documented CSC markers in the HCT116 cell-type by RT-PCR, namely *NANOG, SOX2, OCT4*, and *ALDH1* (Fig. 2B, 2C) [37–39].

Our results demonstrated no significant (*p* > 0.05) alteration in the proportion of CD44^+^ or CD133^+^ in both cell models (data not shown). However, for both HCT116 and HT29 cells, the mean fluorescent intensity (MFI) of CD44 in NDRG1 over-expressing cells was significantly (*p* < 0.001–0.01) lower than NDRG1 Con cells, while for sh NDRG1 cells, CD44 was significantly (*p* < 0.001–0.05) higher compared to sh Con cells (Fig. 2A). On the other hand, CD133 expression was not significantly (*p* > 0.05) altered by NDRG1 over-expression or silencing in HCT116 cells (Fig. 2A). Examining HT29 cells, NDRG1 over-expression significantly (*p* < 0.05) increased CD133 levels, while NDRG1 silencing had no significant (*p* > 0.05) effect on CD133 expression relative to the sh Con. These results indicate that NDRG1 consistently regulates CD44 expression in HCT116 and HT29 cells, while CD133 was not consistently affected in both cell-types.

As shown in Figs. 2B and 2C, NDRG1 over-expression significantly (*p* < 0.05) decreased *OCT4* and *NANOG* mRNA levels, while NDRG1 silencing had no significant (*p* > 0.05) effect on the levels of these transcripts relative to the NDRG1 Con. Examining *SOX2* mRNA expression, NDRG1 silencing significantly (*p* < 0.001) enhanced its expression relative to the sh Con, while NDRG1 over-expression had no significant (*p* > 0.05) effect (Fig. 2B, 2C). No significant (*p* > 0.05) alterations were detected in *ALDH1* mRNA levels upon modulation of NDRG1 expression. Overall, these results suggested that NDRG1 regulates CD44 expression, but other CSC markers were not consistently affected. Hence, in subsequent studies below, only CD44 was examined further.

### NDRG1 inhibits tumorigenic ability of CRC cells *in vivo*

**Figure 3:**
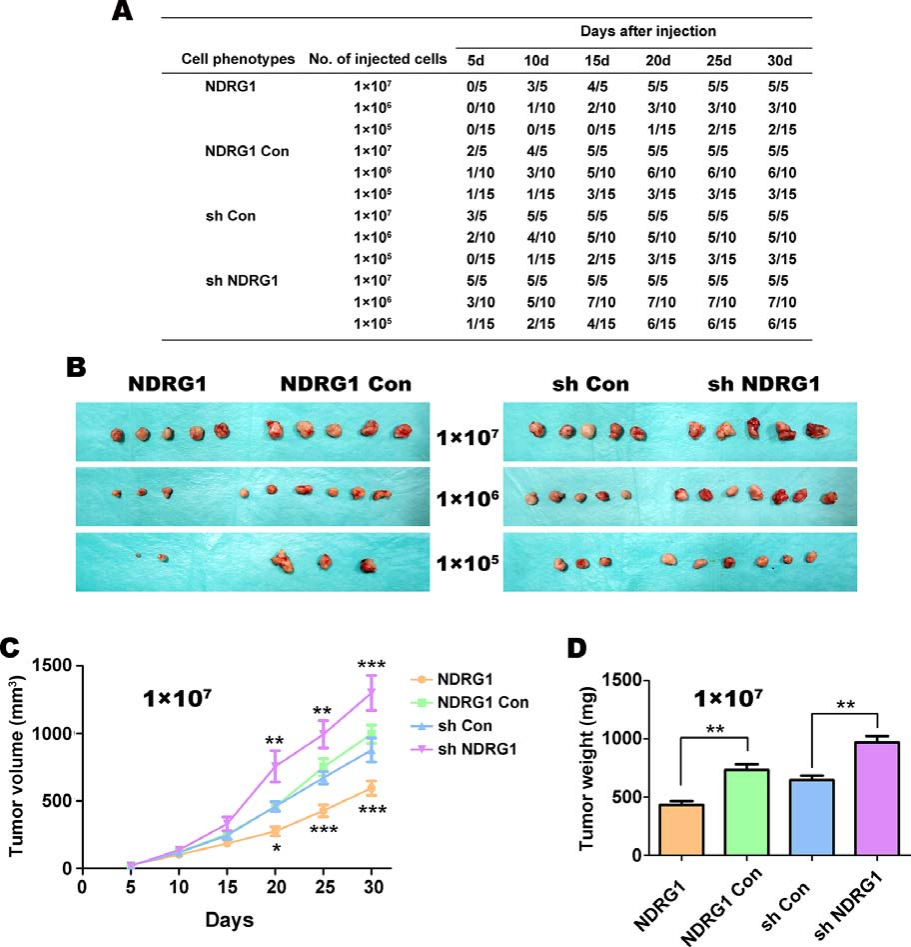
HT29 cells over-expressing NDRG1 are less tumorigenic *in vivo* in nude mice **A.** Tumor formation ability of HT29 cells with NDRG1 over-expression or silencing was monitored every five days up to 30 days after injection into nude mice. Mice inoculated with cells over-expressing NDRG1 levels had lower rates of tumor incidence and tumor formation relative to the vector control or cells with NDRG1 silencing. **B.** Morphological observation of tumors formed after injection of nude mice with HT29 cells that have NDRG1 over-expression or silencing (*i.e*., NDRG1 *vs*. NDRG1 Con, sh Con *vs*. sh NDRG1) when injected *s.c*. at 1 × 10^5^, 1 × 10^6^ and 1 × 10^7^ cells per mouse. **C.** Growth curve of tumors in nude mice (1 × 10^7^ cells per mouse). Tumor diameters were measured every 5 days using Vernier calipers. **D.** Average weight of tumors taken from nude mice after 30 days of growth. All data are shown as mean ± SD (*n* = 5–15 mice/group). *p < 0.05; **p < 0.01; ***p < 0.001.

Any *in vitro* assays used to identify putative “stemness” of cancer cells must be verified by functional assays [40]. In fact, the “gold standard” of stemness is serial transplantation in animal models [41]. Thus, in order to investigate tumorigenicity *in vivo*, 3-week-old male nude mice were injected subcutaneously into the flank with 1 × 10^5^, 1 × 10^6^, or 1 × 10^7^ HT29 cells/mouse (Fig. 3A–3D). Notably, only one cellular dilution was used per mouse. Studies were performed with all 4 clones (*i.e*., NDRG1 Con, NDRG1, sh Con, sh NDRG1) and the growth of the resultant tumor xenografts was monitored every 5 days for 30 days using Vernier calipers (*n* = 5–15 mice/group).

The injection of 1 × 10^7^ cells/mouse yielded tumors in all mice after 5–20 days irrespective of the clone used (Fig. 3A). Interestingly, 20 days after injection of nude mice with 1 × 10^5^ sh NDRG1 cells, 6/15 tumors were observed, compared to only 1/15 from the NDRG1 over-expressing cells (Fig. 3A). However, the NDRG1 over-expressing cells still gave rise to macroscopic tumors (Fig. 3B), although this occurred later than with the other groups, particularly with lower cellular dilutions (Fig. 3A).

Examining tumor volume over 30 days, our results showed that tumors derived from injecting 1 × 10^7^ cells grew significantly (*p* < 0.001–0.05) slower in the NDRG1 over-expressing group than those in the NDRG1 Con and all other groups (Fig. 3C). Conversely, tumors derived from the sh NDRG1 cells grew markedly and significantly (*p* < 0.001–0.01) faster than their relevant sh Con cells (Fig. 3C).

For the mice injected with 1 × 10^7^ tumor cells, the mice were sacrificed after 30 days and the subcutaneous tumors then weighed (Fig. 3D). The average weight of the tumors derived from the NDRG1 over-expressing cells was significantly (*p* < 0.01) less than their relevant NDRG1 Con tumors. In contrast, the weight of tumors from sh NDRG1 cells was significantly (*p* < 0.01) greater than those derived from the sh Con cells (Fig. 3D). Together, these results in Figure 3 indicate that NDRG1 plays an important role in the tumorigenicity of HT29 CRC cells *in vivo*.

### NDRG1 inhibits nuclear β-catenin expression and activation of β-catenin signaling

It has been reported that WNT signaling plays an important role in the maintenance of the CSC pool and prevention of cellular differentiation [42]. In our previous studies, NDRG1 was demonstrated to increase β-catenin expression at the cell membrane, while decreasing its nuclear expression and TCF/LEF signaling [18, 22]. Hence, it can be speculated that NDRG1 may inhibit stem CSC-like properties through down-regulation of nuclear β-catenin expression and signaling.

**Figure 4:**
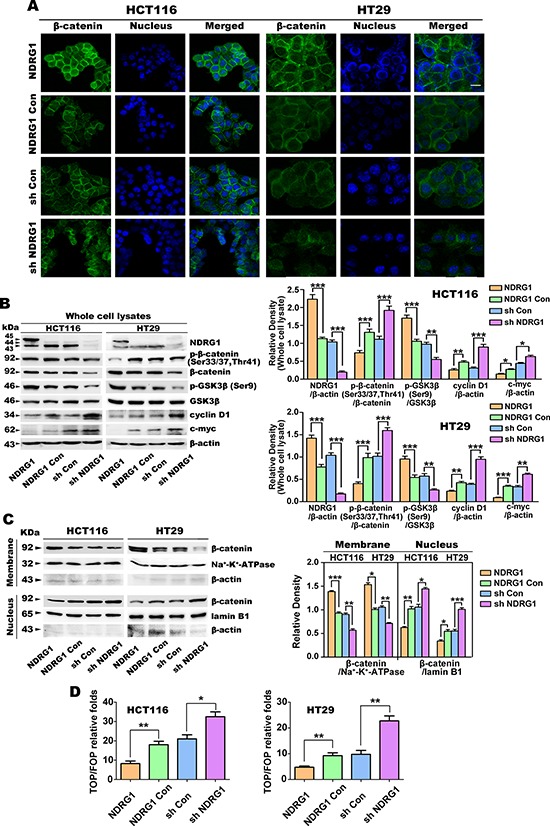
Over-expression of NDRG1 down-regulates nuclear β-catenin expression in HCT116 and HT29 cells **A.** Immunofluorescent staining of β-catenin in human HCT116 and HT29 CRC cells (green: β-catenin; blue: nuclear (DAPI) staining; scale bar, 20 μm). **B.** The protein expression of p-β-catenin (Ser33/37, Thr41), total β-catenin, p-GSK3β, GSK3β, cyclin D1 and c-myc, in whole cell lysates of HCT116 or HT29 cells. β-actin was used as the loading control for the whole cell lysate. Densitometry represents the expression of the proteins relative to β-actin. **C.** The expression of total β-catenin in the membrane and nuclear fractions of HCT116 and HT29 cells. Na^+^-K^+^-ATPase and lamin B1 were used as the loading controls for the membrane and nuclear fractions, respectively. Densitometry represents the expression of membrane and nuclear β-catenin compared with the respective loading controls. **D.** TOP/FOP Flash assay was used to assess the transcriptional activity of nuclear β-catenin in HCT116 and HT29 cells. All data are shown as mean ± SD (*n* = 3) *p < 0.05; **p < 0.01; ***p < 0.001.

In order to examine this hypothesis, we firstly performed immunofluorescence and immunoblotting to detect β-catenin expression in different cellular compartments of HCT116 and HT29 cells. As shown in Fig. 4A, NDRG1 over-expression in both HCT116 and HT29 cells enhanced β-catenin expression at the cell membrane. On the other hand, sh NDRG1 cells displayed less membrane-bound β-catenin expression relative to the sh Con (Fig. 4A).

Following on from these immunofluorescence results, immunoblotting was then performed to assess the expression of NDRG1, p-β-catenin (Ser33/37, Thr41), total β-catenin, p-GSK3β (Ser9), total GSK3β, cyclin D1 and c-myc in whole cell lysates (Fig. 4B). As demonstrated in our previous studies in multiple cell-types [18, 21, 22, 43], NDRG1 appeared as multiple bands between approximately 43 and 45 kDa in both HCT116 and HT29 cells (Fig. 4B). In NDRG1 over-expressing cells, the top band at approximately 45 kDa represents exogenous protein with the FLAG-tag [18, 21]. As expected, NDRG1 expression was significantly (*p* < 0.001) greater in NDRG1 cells relative to NDRG1 Con cells, and conversely, NDRG1 in sh NDRG1 cells was significantly (*p* < 0.001) less than that in sh Con cells (Fig. 4B).

Next, the effect of NDRG1 expression on the phosphorylation of β-catenin at Ser33/37 and Thr41 was assessed (Fig. 4B), as this modification leads to degradation of the protein [44–46]. In both the HCT116 and HT29 cell-types, relative to the NDRG1 Con, NDRG1 over-expression also resulted in a significant (*p* < 0.001) decrease in the ratio of β-catenin phosphorylation at Ser33/37,Thr41 to total β-catenin (Fig. 4B). In contrast, NDRG1 silencing in both cell-types relative to the sh Con cells, led to a significant (*p* < 0.001) increase in the ratio of β-catenin phosphorylation at Ser33/37, Thr41 to total β-catenin. Notably, total β-catenin increased slightly, but was not significantly (*p* > 0.05) altered in NDRG1 over-expressing cells relative to NDRG1 Con cells. However, total β-catenin expression did decrease significantly (*p* < 0.001) in sh NDRG1 clones for both the HCT116 and HT29 cell-types (Fig. 4B). These results regarding the effects of NDRG1 on β-catenin expression were similar to those reported previously by our laboratory [22].

As the phosphorylation of GSK3β at Ser9 inactivates this protein leading to a decrease in phosphorylation of β-catenin at Ser33/37,Thr41 and the inhibition of β-catenin degradation [47], we next examined the effect of NDRG1 over-expression and silencing on GSK3β phosphorylation at Ser9 (Fig. 4B). Using both HCT116 and HT29 cells, NDRG1 over-expression significantly (*p* < 0.001) increased the ratio of GSK3β phosphorylation at Ser9 to total GSK3β. In contrast, silencing NDRG1 significantly (*p* < 0.01) decreased this ratio. However, there was no significant (*p* > 0.05) difference in total GSK3β between the NDRG1 and NDRG1 Con clones, and also between the sh Con and sh NDRG1 clones. These observations suggest that NDRG1 can promote GSK3β phosphorylation at Ser9, leading to inhibition of β-catenin phosphorylation at Ser33/37,Thr4. Hence, these effects prevent the subsequent degradation of β-catenin, as shown previously by our laboratory [22].

Considering the alterations in β-catenin described above, the protein expression of its downstream targets, cyclin D1 and c-myc, were also examined (Fig. 4B). Both of these targets were also significantly (*p* < 0.001–0.05) down-regulated in HCT116 and HT29 cells by the over-expression of NDRG1 relative to the NDRG1 Con. Conversely, NDRG1 silencing resulted in a significant (*p* < 0.001–0.05) increase of cyclin D1 and c-myc in both these cell-types relative to the sh Con (Fig. 4B).

The results above indicated potential regulation of β-catenin by NDRG1. Hence, the subcellular distribution of β-catenin was then examined in the membrane and nuclear fractions (Fig. 4C), as its re-distribution plays a critical role in its function [48, 49]. Upon NDRG1 over-expression in both cell-types, there was a significant (*p* < 0.001–0.05) increase in membrane β-catenin expression and a significant (*p* < 0.01–0.05) decrease in nuclear β-catenin levels. In contrast, NDRG1 silencing had the opposite effect, leading to a significant (*p* < 0.01) decrease in the membrane β-catenin level and a significant (*p* < 0.001–0.05) increase in the nuclear β-catenin fraction relative to the sh Con (Fig. 4C). These effects of NDRG1 on β-catenin expression were not observed (*p* > 0.05) for the plasma membrane protein, Na^+^-K^+^-ATPase, nor the nuclear protein, lamin B1 (Fig. 4C). Hence, the effect of NDRG1 on β-catenin levels was not a general effect observed on other plasma membrane or nuclear proteins. The levels of β-actin in both the membrane and nuclear fractions were low and generally inconsistent (probably due to the preparation of these subcellular fractions), and hence, Na^+^-K^+^-ATPase and lamin B1 were used in preference as loading controls (Fig. 4C).

Finally, we assessed the transcriptional activity of nuclear β-catenin in transfected HCT116 and HT29 cells using the TOP/FOP Flash assay [22]. As shown in Fig. 4D, NDRG1 over-expression significantly (*p* < 0.01) decreased β-catenin transcriptional activity relative to the NDRG1 Con. On the other hand, silencing of NDRG1 in both HCT116 and HT29 cells resulted in significantly (*p* < 0.01–0.05) greater β-catenin transcriptional activity than the sh Con groups. Collectively, NDRG1 over-expression reduced nuclear β-catenin expression and inhibited its downstream signaling, while NDRG1 silencing had the opposite effect.

### Down-regulation of β-catenin reverses the CSC phenotype and tumorigenesis caused by NDRG1 silencing

**Figure 5:**
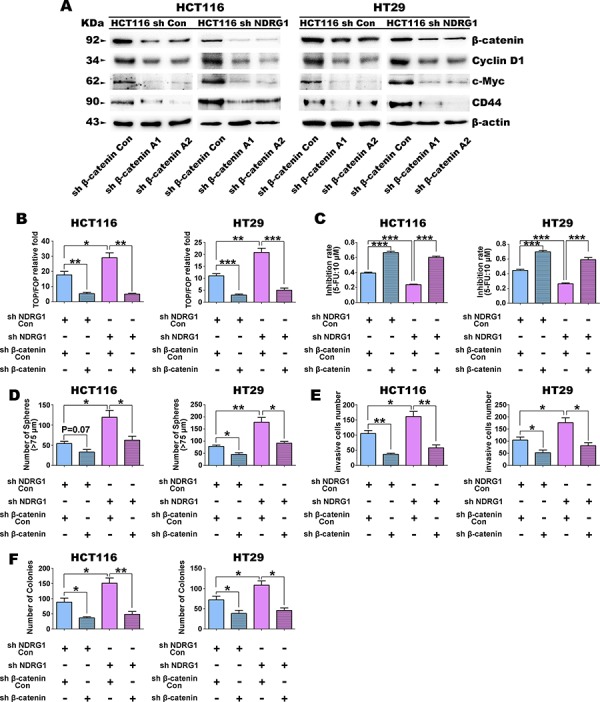
Down-regulation of β-catenin reverses the stem cell-like phenotypes in HCT116 and HT29 cells caused by NDRG1 silencing **A.** The expression of β-catenin, cyclin D1, as well as the CSC marker, CD44, after shRNA transfection targeting β-catenin in sh Con or sh NDRG1 clones of HCT116 or HT29 cells. **B.** Silencing of β-catenin decreases TOP/FOP transcription activity in HCT116 and HT29 cells with NDRG1 silencing. **C.** Silencing of β-catenin increases sensitivity to 5-fluorouracil (5-FU: 10 μmol/L) in HCT116 and HT29 cells with NDRG1 silencing. **D.** Silencing of β-catenin in HCT116 or HT29 cells decreases sphere formation (>75 μm) ability. **E.** Down-regulation of β-catenin inhibits the invasive ability of HCT116 and HT29 cells with NDRG1 silencing. **F.** Silencing of β-catenin decreases colony formation activity in HCT116 and HT29 cells with NDRG1 silencing. All data are shown as mean ± SD (*n* = 3). *p < 0.05; **p < 0.01; ***p < 0.001.

Our studies above in Figure 1–4 demonstrate that NDRG1 silenced cells express higher nuclear β-catenin levels and show more CSC traits and tumorigenic ability. To investigate whether β-catenin down-regulation can reverse CSC properties and tumorigenesis caused by NDRG1 silencing, two different short hairpin RNAs (shRNAs), designated A1 and A2, were used to silence β-catenin expression in sh NDRG1 and sh Con cells (Fig. 5A). In fact, both shRNAs caused a significant (*p* < 0.001) decrease in total β-catenin expression relative to the control in both HCT116 and HT29 cells (Fig. 5A). Considering the successful β-catenin silencing, the expression of its target genes, namely cyclin D1 and c-myc, as well as the CSC marker CD44, were examined and found to be significantly (*p* < 0.05) decreased (Fig. 5A). Moreover, β-catenin transcriptional activity was also significantly (*p* < 0.001–0.01) inhibited in both HT29 and HCT116 cells in response to β-catenin silencing, as demonstrated by the TOP/FOP Flash assay (Fig. 5B). In fact, while sh NDRG1/sh β-catenin Con cells were found to have significantly (*p* < 0.01–0.05) higher β-catenin transcriptional activity than the sh NDRG1 Con/sh β-catenin Con cells in both HCT116 and HT29 cell-types, the β-catenin shRNA (*i.e*., sh β-catenin) was still able to markedly and significantly (*p* < 0.001–0.01) inhibit this effect in both sh NDRG1 Con and sh NDRG1 cells (Fig. 5B).

We further assessed the effect of β-catenin down-regulation on the stem-like phenotype of sh NDRG1 HT29 and HCT116 cells, as well as their relevant controls (Fig. 5C–5E). We firstly elucidated the role of β-catenin in chemoresistance in these cell-types (Fig. 5C). Cells were cultured with 10 μM 5-FU and the inhibition of cellular proliferation was then calculated after a 36 h incubation (Fig. 5C). Compared with the sh β-catenin Con, silencing of β-catenin significantly (*p* < 0.001) increased the sensitivity of both sh NDRG1 Con or sh NDRG1 cells to 5-FU by approximately 2-fold (Fig. 5C), suggesting that β-catenin plays a crucial role in resistance to chemotherapy.

The ability of the HCT116 and HT29 clones to form spheres was then assessed (Fig. 5D). These results demonstrated that the number of spheres (≥75 μm) formed by sh NDRG1 Con/sh β-catenin Con cells was reduced (*p* = 0.07) or significantly (*p* < 0.05) reduced when β-catenin was silenced. This effect of β-catenin silencing on sphere formation was also observed for sh NDRG1/sh β-catenin Con cells, where a significant (*p* < 0.05) decrease was identified in the sh NDRG1/sh β-catenin cells (Fig. 5D).

Next, invasion assays were performed to detect whether silencing of β-catenin could affect the invasive ability of these cells *in vitro*. As is shown in Fig. 5E, the number of invading HCT116 and HT29 cells with silenced β-catenin (*i.e*., sh NDRG1 Con/sh β-catenin) was significantly (*p* < 0.01–0.05) less than control cells treated with sh NDRG1 Con/sh β-catenin Con. Hence, decreasing β-catenin expression reduces invasion relative to the control. Studies examining HCT116 and HT29 cells treated with sh NDRG1/sh β-catenin Con led to a significant increase (*p* < 0.05) in invasion relative to the sh NDRG1 Con/sh β-catenin Con cells (Fig. 5E), demonstrating the role of NDRG1 in suppressing invasion (as also shown in Fig. 1C).

Studies then assessed the effect on invasion of silencing both NDRG1 and β-catenin using sh NDRG1 and sh β-catenin in HCT116 and HT29 cells (Fig. 5E). In these investigations, silencing the expression of both these proteins (*i.e*., sh NDRG1/sh β-catenin) significantly (*p* < 0.01–0.05) decreased invasion relative to these cells silenced with NDRG1 alone (*i.e*., sh NDRG1/sh β-catenin Con; Fig. 5E). These data again demonstrate the importance of β-catenin expression in promoting invasion.

Finally, assays examining soft-agar colony formation (Fig. 5F) showed that down-regulation of β-catenin (sh β-catenin) could reduce the number of colonies to less than 50 per well, which was significantly (*p* < 0.05) less than their control groups in both cell lines.

Interestingly, in all the assays examined above (see Fig. 5B–5F), there were no significant (*p* > 0.05) differences between the sh Con and sh NDRG1 cells once β-catenin was silenced. These results suggest that the low expression of nuclear β-catenin may inhibit the CSC phenotype *in vitro*.

Collectively, nuclear β-catenin plays a vital role in maintaining stem-like traits in these cancer cell lines. The silencing of NDRG1, which increases nuclear β-catenin and its down-stream target CD44 [50], results in increased stem-like traits, while the down-regulation of β-catenin can reverse these properties.

### There is a negative correlation between NDRG1 and nuclear β-catenin and also NDRG1 and CD44 expression in CRC clinical specimens

**Figure 6:**
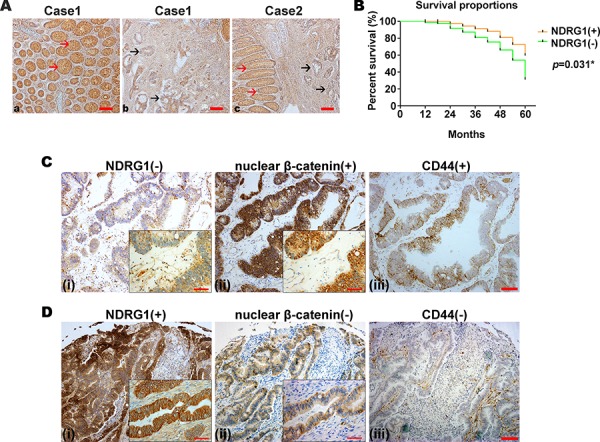
NDRG1 is negatively associated with tumorigenesis in CRC clinical specimens and with nuclear β-catenin and CD44 expression **A.** Immunohistochemistry of colon tissue samples demonstrating that NDRG1 is highly expressed in the adjacent normal intestinal glands compared to CRC tissues in two representative cases (red arrows: normal glands; black arrows: CRC regions). Magnification: 200×. **B.** Survival of CRC patients with tumors positive or negative for NDRG1 for periods up to 60 months. **C.** Immunohistochemistry of CRC tissue samples from a representative patient with: (i) low NDRG1 (*i.e*., NDRG1(−)); (ii) high nuclear β-catenin (*i.e*., nuclear β-catenin(+)); and (iii) high total CD44 levels (*i.e*., CD44(+)). **D.** Immunohistochemistry of CRC tissue samples from a representative patient with: (i) high NDRG1 (*i.e*., NDRG1(+)); (ii) low nuclear β-catenin (*i.e*., nuclear β-catenin(−)); and (iii) low total CD44 levels (*i.e*., CD44(−)). Magnification: 200 ×. Scale Bar = 200 μm. Insets represent magnified images of each section (400x). Scale Bar = 100 μm.

In order to investigate the correlation of NDRG1, nuclear β-catenin and CD44 in clinical CRC specimens, we first examined the expression of NDRG1, nuclear β-catenin and CD44 in 116 cases of CRC using a tissue microarray (Fig. 6). As shown in Fig. 6A and Supplementary Table 1, comparing normal intestinal glands (red arrows) to CRC regions (black arrows), the normal glands expressed higher NDRG1 levels. Of the 116 patient samples examined, 66 (56.9%) of the normal tissues were positive for NDRG1 (immunoreactive score (IRS) > 4; see *Materials and Methods: Clinical Colorectal Specimens and Immunohistochemistry*), while 39 (33.6%) of the CRC cases expressed significantly (*p* = 0.001) positive NDRG1 (Supplementary Table 1). Also, compared with NDRG1 negative CRC cases, NDRG1 positive CRC cases had a significantly (*p* = 0.031) better prognosis (Fig. 6B).

It is notable that in the CRC tissues examined, NDRG1 was mainly localized in the cytoplasm or on the membrane, with only rare nuclear expression, while β-catenin was mainly expressed in the cytoplasm and nucleus (see magnified insets; Fig. 6C, 6D). To demonstrate typical staining, Figure 6C shows a CRC tissue sample from a representative patient with relatively low NDRG1 (*i.e*., NDRG1(−); Fig. 6C(i)) and relatively high nuclear β-catenin (*i.e*., nuclear β-catenin(+); Fig. 6C(ii)) and total CD44 (*i.e*., CD44(+); Fig. 6C(iii)). On the other hand, Figure 6D is from a CRC tissue sample from a patient with high NDRG1 (*i.e*., NDRG1(+); Fig. 6D(i)) and low nuclear β-catenin (*i.e*., nuclear β-catenin(−); Fig. 6D(ii)) and total CD44 (*i.e*., CD44(−); Fig. 6D(iii)).

**Figure 7:**
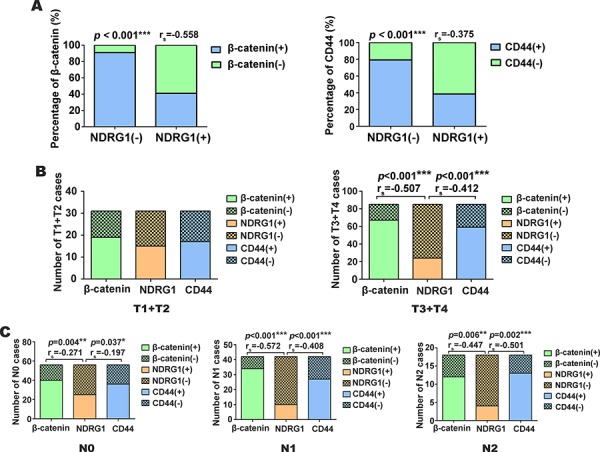
NDRG1 is negatively correlated with CRC tumor invasion and lymphatic metastasis as judged by immunohistochemistry in tissue samples **A.** Percentage of nuclear β-catenin and total CD44 expression in NDRG1 negative or positive CRC cases as judged by immunohistochemistry. **B.** The correlation between nuclear β-catenin, total NDRG1 and CD44 expression as a function of different tumor invasion categories (*i.e*., T1+T2 and T3+T4) in CRC cases. **C.** The correlation between nuclear β-catenin, total NDRG1, and total CD44 expression as a function of different lymphatic metastasis categories (*i.e*., N0, N1 and N2) in CRC cases. The invasion and lymphatic metastasis was assessed by staging criteria according to 7th Edition of the American Joint Committee on Cancer (AJCC) [50] (see text for further details). *p < 0.05; **p < 0.01; ***p < 0.001.

Intriguingly, analysis of all 116 CRC patient cases demonstrated the regions with high nuclear β-catenin and total CD44 expression were well correlated to negative NDRG1 expression, while those samples with low β-catenin and total CD44 expression were correlated to high NDRG1 expression. In fact, in 77 NDRG1 negative cases, 70 cases (*i.e*., 91%) had prominent nuclear β-catenin expression, while among 39 NDRG1 positive cases, 23 cases (*i.e*., 59%) were β-catenin negative, (Supplementary Table 2; Fig. 7A). Hence, NDRG1 was negatively correlated with nuclear β-catenin expression (Fig. 7A; *p* < 0.001; *r_s_* = − 0.558).

Assessing the relationship between CD44 expression and NDRG1, in the 77 NDRG1 negative cases, 61 cases (*i.e*., 78%) were CD44 positive (Supplementary Table 2; Fig. 7A). On the other hand, in 39 NDRG1 positive cases, 24 (*i.e*., 62%) were CD44 negative (Supplementary Table 2; Fig. 7A, *p* < 0.001, *r_s_* = −0.375).

Considering the observed comparatively low expression of NDRG1 in CRC, the relationship between NDRG1 expression and the pathological features of these tumors was then further investigated. As shown in Supplementary Table 3, NDRG1 expression was negatively related to tumor invasion (*p* = 0.022) and lymphatic metastasis (*p* = 0.011). There was no significant (*p* > 0.05) relationship between NDRG1 expression and other parameters, including: gender, age, or tumor location (Supplementary Table 3).

We further calculated the correlation coefficient between NDRG1 and β-catenin expression, and between NDRG1 and CD44 expression in CRC cases demonstrating different extents of invasion and lymphatic metastasis. The invasion and lymphatic metastasis was assessed in terms of staging criteria according to the 7^th^ Edition of the American Joint Committee on Cancer (AJCC; Tumor size, Lymph nodes involved, Metastases (TNM) staging) [51]. The CRC invasion was presented as T (Tumor), including:- T1: limited to mucosa and submucosa; T2: extension into the thick muscular layer; T3: invasion to subserosa or entire colon or rectum wall; and T4: invasion of adjacent structures. The lymphatic metastasis was presented as:- N (Node), including: N0: no involved lymph nodes; N1: fewer than 4 regional nodes involved; and N2: four or more lymph nodes involved.

As demonstrated in Fig. 7B, in less invasive cases (T1+T2), there was no significant (*p* > 0.05) correlation detected between NDRG1, β-catenin and CD44 expression. However, in advanced invasive cases (T3+T4), NDRG1 had significant negative correlations with β-catenin and CD44 expression (*p* < 0.001 *r_s_* = −0.507, *p* < 0.001 *r_s_* = −0.412, respectively). In the lymph node metastasis assessment (Fig. 7C), there was a less negative correlation between NDRG1, β-catenin and CD44 expression (*p* = 0.004 *r_s_* = −0.271; *p* = 0.037 *r_s_* = −0.197, respectively) in the N0 cases. In the N1 cases, more NDRG1 negative (*i.e*., IRS < 4) cases were β-catenin (*p* < 0.001 *r_s_* = −0.572) and CD44 (*p* <0.001 *r_s_* = −0.408) positive (Fig. 7C). While in the N2 cases, there was also a negative correlation between NDRG1, β-catenin and CD44 expression (*p* = 0.006 *r_s_* = −0.447; *p* < 0.002 *r_s_* = −0.501 respectively).

Taken together, these data in Supplementary Table 4 and Figs. 7B and 7C indicate that with the progression of tumor invasion and the increase of lymph node metastases, NDRG1 has a stronger negative association with nuclear β-catenin and CD44 expression. On the other hand, nuclear β-catenin has a positive correlation with CD44 expression in CRC.

## DISCUSSION

Increasing evidence demonstrates that NDRG1 functions as a metastasis suppressor and potentially could be a novel indicator for CRC prognosis [16, 25, 52]. Although NDRG1 has been defined as a metastasis suppressor gene, it is of interest that *NDRG1* mRNA expression was found to be up-regulated in colorectal cancer data from the Cancer Genome Atlas (TCGA) [53]. In addition, in some tumor-types (*e.g*., hepatocellular carcinoma), NDRG1 was associated with more aggressive phenotypes and a poorer prognosis [27]. These paradoxical effects of NDRG1 may due to potential post-translational regulation events that have been shown to affect NDRG1 function and could be mediated by genetic alterations that occur in different cancer-types [19, 22].

In an earlier study, we demonstrated that NDRG1 can inhibit TGF-β-induced EMT *via* its inhibitory effects on EMT transcription factors, such as Snail and Slug, which play a role in regulating cell invasion and metastasis [18]. We also demonstrated that NDRG1 decreased the nuclear accumulation of β-catenin through down-regulation of its nuclear co-translocation protein, PAK4 [22]. Studies performed by Liu *et al*. also showed that NDRG1 interacts with the Wnt receptor, LRP6, which results in inhibition of the Wnt signaling pathway and its down-stream effects on promoting metastasis [54].

Many signaling pathways, including Wnt, Hedgehog and Notch, are commonly involved in maintaining the EMT and CSCs [55]. Recently, several studies have shown that the EMT could enrich cells with CSC properties, and it is notable that CSCs exhibit a mesenchymal phenotype [56, 57]. However, an investigation by Ocana *et al*. showed that the features of the newly discovered EMT activator, Prrx1, surprisingly suppressed stem-like traits including mammosphere formation and self-renewal capacity [14]. Hence, the results from this previous investigation suggested that EMT and stem-like properties are not necessarily linked [14]. The specific molecular relationship between the EMT and acquisition of CSCs properties is still unknown and remains to be determined [15, 58].

The Wnt/β-catenin pathway mediates a wide variety of processes, including cell proliferation, migration, differentiation, adhesion and apoptosis [59]. Interestingly, β-catenin can function as both an oncogene and metastasis suppressor, with its activity being dependent on its localization [59, 60]. For instance, when expressed at the cell membrane, β-catenin binds to E-cadherin to form the adherens junction complex which inhibits metastasis [60]. However, upon activation of the Wnt pathway in CRC, β-catenin associates with adenomatous polyposis coli (APC) and GSK3β in the cytosol, leading to its nuclear translocation [61]. In the nucleus, β-catenin associates with TCF/LEF factors to regulate the expression of Wnt downstream genes (*e.g*., *cyclin D1* and *c-myc*), contributing to tumorigenesis [62].

Our data herein indicate that NDRG1 expression can inhibit stem cell-like traits and tumorigenesis in CRC both *in vitro* and *in vivo* which could be linked to its ability to act as a negative EMT regulator [18]. Conversely, down-regulation of NDRG1 increased β-catenin nuclear accumulation and activation of its signaling. Importantly, silencing of β-catenin could reverse the CSC-like properties and tumorigenesis caused by NDRG1 silencing, highlighting the importance of β-catenin in this process. Indeed, Wnt/β-catenin signaling has been described to be important for maintaining stem-like traits [63]. For instance, Wnt/β-catenin signaling is necessary for maintenance of stem cells in the intestinal crypts [64]. Moreover, Wnt inhibitors reduce stem cell-like characteristics in prostate cancer cells, whereas Wnt3a stimulates sphere formation and self-renewal [65].

In this study, although NDRG1 over-expressing HT29 and HCT116 cells showed a slight, but not significant (*p* > 0.05) increase in total β-catenin expression (Fig. 4B), these cells displayed decreased nuclear β-catenin (Fig. 4C) and transcriptional activity (Fig. 4D). These observations can be explained by the fact that NDRG1 down-regulates PAK4, which functions as “a partner” for β-catenin nuclear translocation, preventing its entrance into the nucleus [22]. Further, it is was notable that NDRG1 expression inhibits the degradation of β-catenin by decreasing the ratio of its phosphorylation at Ser33,37/Thr41 (Fig. 4B), resulting in the increase in total β-catenin observed. Notably, we demonstrated that NDRG1 over-expression induces two effects: (1) greater β-catenin is present in the cell membrane fraction (Fig. 4C) which is critical for cell-cell adhesion and reducing metastasis [66]; and (2) less β-catenin is present in the nuclear fraction (Fig. 4C) that reduces transcription of oncogenic genes such as *c-myc* and *cyclin D1*, as shown by the TOP/FOP Flash assay (Fig. 4D). These results confirm our previous studies examining the role of NDRG1 over-expression on β-catenin localization and its anti-metastatic and anti-oncogenic effector roles [22].

In our study, down-regulation of β-catenin in CRC cells with NDRG1 silencing resulted in the inactivation of Wnt signaling, down-regulation of CD44, depression of CSC properties and tumorigenesis (Fig. 5). Moreover, although NDRG1 did not reduce the proportion of CD44^+^ or CD133^+^ cells, it significantly decreased CD44 expression, which could potentially contribute to the loss of CRC stem-like traits. Notably, both CD44 and CD133 have been reported as markers of CSCs in human colorectal cancer [34, 67, 68]. However, Shmelkov *et al.* and LaBarge and Bissell have challenged the view that CD133 is a marker for CSCs in colorectal cancer [69, 70]. One study performed by Du *et al*. demonstrated that CD44, rather than CD133, is more suitable as a marker of colorectal CSCs [71] and this was consistent with the results of the present investigation. CD44 is of functional importance for maintaining the stem cell-like phenotype and for supporting cancer cell expansion [71–73]. Furthermore, CD44 has been shown to be a downstream target of the β-catenin/TCF signaling pathway [74–76]. Elevated CD44 has been correlated with the activation of β-catenin in Twist over-expressing cells, while treatment with Wnt3a could further induce the activation of β-catenin and the induction of CD44 [59].

The immunohistochemistry results performed in this investigation demonstrated that the expression of NDRG1 is weaker in CRC tissues than in the corresponding normal mucosa. Moreover, the current study showed that NDRG1 positive CRC cases had a better prognosis when compared to NDRG1 negative cases (Fig. 6B). The regions where NDRG1 levels were low were also associated with higher nuclear β-catenin expression, and *vice versa*. There was also a close negative correlation between NDRG1 and nuclear β-catenin and also NDRG1 and CD44 expression in CRC patients with tumor invasion and metastasis to lymph nodes. In agreement with our investigation, studies performed by Strzelczyk et al., and Manish et al., also demonstrated that NDRG1 expression was negatively correlated with colorectal cancer progression and prognosis [77, 78].

In summary, the present study indicates that NDRG1 attenuates CSC characteristics and tumorigenesis of CRC *in vitro* and *in vivo*. Moreover, NDRG1 could function as a metastasis suppressor for CRC through inactivation of β-catenin signaling and down-regulation of CD44. Identification of the role of NDRG1 as a metastasis suppressor provides a novel diagnostic biomarker and a therapeutic target for the treatment of CRC.

## MATERIALS AND METHODS

### Cell culture and antibody reagents

The human colorectal cancer cell lines, HT29 and HCT116, were purchased from Shanghai Institute of Biochemistry and Cell Biology (Shanghai, China) and stored in the Key Laboratory of Cell Differentiation and Apoptosis of the National Ministry of Education (Shanghai, China). NDRG1 over-expressing and silenced clones of the colorectal cancer cell lines, HT29 and HCT116, were constructed, as previously described [18]. Both HT29 and HCT116 cells were cultured in McCoy's 5A medium (Gibco, USA) with 10% fetal bovine serum (FBS; Gibco, USA). Cells were cultured in humidified air with 5% CO_2_ at 37°C.

The primary antibodies used were as follows: goat anti-NDRG1, mouse anti-cyclin D1, rabbit anti-lamin B1, mouse anti-Na^+^-K^+^ATPase β2 (Abcam, USA); rabbit anti-β-catenin, mouse anti-CD44 (Cell Signaling Technology, Inc., USA); mouse anti-c-Myc, mouse anti-β-actin (Santa Cruz Biotechnology, USA); Phycoerythrin (PE)-labeled anti-human CD44 (BD Pharmingen™, USA) and PE-labeled anti-human CD133/1 (AC133) (Miltenyi Biotec, Germany).

### Tumor sphere formation assay

The cells were detached from culture flasks with 0.25% trypsin and suspended in sphere formation medium (*i.e*., 50 mL of DMEM/F12 containing 100 mg/mL EGF, 100 mg/mL bFGF, and 1 mL of B-27^®^ Supplement; Gibco, USA). Then cells were filtered into a single-cell suspension and seeded (10,000 cells/mL) in an ultra-low attachment 24-well plate (Corning, USA). The cells were cultivated for 12 to 14 days and the spheres were observed under a phase-contrast microscope (Olympus, Japan). In order to assess the self-renewal ability of these cells, formed spheres were separated into a single cell suspension and then again seeded into serum-free culture and the sphere formation rate of “offspring” cells was calculated.

### Cell invasion assay

Cell invasion was assessed using transwell chambers (8.0 μm pore size, Corning, USA) with Matrigel (BD Bioscience, USA) according to the manufacturer's protocols. Then 8 × 10^4^ cells in 200 μL of serum-free McCoy's 5A media were placed in the top chamber and 600 μL of 10% FBS-containing medium was placed into the bottom chamber. After incubation for 24 h/37°C, the cells that did not invade to the lower side of the chamber were removed from the top side. The chambers were then stained with crystal violet and photographed.

### Soft agar colony formation assay

The soft agar assay was performed to determine transformation and anchorage- independent growth [79]. In this assay, 6-well plates were covered with two layers of low melting-point agarose. The bottom layer consisted of 1.3% agar in 1.5 mL of DMEM with 20% FBS. The HT29 and HCT116 cells (2 × 10^3^/well) were mixed in the top layer containing 0.7% agar in the same medium as the bottom. Cells were cultured for 20 days/37°C and colonies were counted and photographed.

### Chemotherapy sensitivity assay

As 5-FU (5-fluorouracil) is one of the widely used chemotherapeutic drugs for CRC treatment [80], we assessed the sensitivity of the HT29 and HCT116 cells to this agent. The effect of NDRG1 expression on the cellular sensitivity to 5-FU was measured by the Cell Counting Kit-8 (CCK8) assay (Dojindo, Japan). Briefly, 5-FU (Sigma-Aldrich, USA) was used at seven different concentrations (*i.e*., 0.1, 1, 5, 10, 50, 100 and 200 μM) in McCoy's 5A medium, then the cells were seeded in this medium at a density of 3000 cells/well in 96-well plates and cultured for 36 h/37°C. Then, 10 μL of CCK8 was added to each well and the absorption value (A) was detected at 450 nm with a UV-Vis spectrophotometer. The inhibition rate was calculated as: (Acontrol−Aexperiment)/Acontrol × 100%.

### Flow cytometric analysis

Cultured cells were detached and washed twice with PBS (5 min at 1000 rpm/min at room temperature) and re-suspended in 100 μL PBS. PE-labeled anti-human CD44 and PE-labeled anti-human CD133/1(AC133) were added respectively, and incubated for 20 min at room temperature in the dark. Cells were washed twice with PBS and then the expression of CD44 and CD133 was detected by flow cytometry.

### RNA extraction, RT-PCR and real-time RT-PCR

Total RNA was extracted from cells using TriPure Isolation Reagent (Roche, Basel, Switzerland) according to the manufacturer's instructions. RT-PCR was performed by standard procedures [81] using the primers in Table 1. Then 1 μg of total RNA was subjected to a reverse transcriptase reaction to generate cDNA. RT-PCR was conducted for cDNA amplification and β-actin was used as an internal control. The RT-PCR cycling program was as follows: pre-denaturation at 95°C for 15 min, followed by 30 cycles of denaturation at 94°C for 15 s, annealing at 55°C for 30 s, and elongation at 70°C for 30 s.

**Table 1:** Primers for amplification of genes used in this study

Primer name	Oligonucleotides (5′–3′)		Product size (bp)
	Forward sequence	Reverse sequence	
NDRG1	CTCCTGCAAGAGTTTGATGTCC	TCATGCCGATGTCATGGTAGG	127
OCT4	CTGGGTTGATCCTCGGACCT	CCATCGGAGTTGCTCTCCA	243
NANOG	AAGGTCCCGGTCAAGAAACAG	CTTCTGCGTCACACCATTGC	237
SOX2	TGGACAGTTACGCGCACAT	CGAGTAGGACATGCTGTAGGT	215
ALDH1	GCACGCCAGACTTACCTGTC	CCTCCTCAGTTGCAGGATTAAAG	129
β-actin	CATGTACGTTGCTATCCAGGC	CTCCTTAATGTCACGCACGAT	250

### Western blotting

Western analysis was performed using standard methods [18]. Briefly, HT29 and HCT116 cells were cultured and lysed in RIPA buffer containing protease inhibitors. Nuclear and membrane components were extracted respectively using NE-PER Nuclear and Cytoplasmic Extraction Reagents (Thermo Scientific, USA) and Proteo Extract@ Native Membrane Proteome Extraction Kit (Merck, Germany). Equal amounts of total proteins (or different components) were loaded and separated on a 10% SDS-PAGE gel, and then transferred to PVDF membranes. After blocking in 50 g/L non-fat milk in TBST (20 mmol/L Tris-HCl, 137 mmol/L NaCl, 1 g/L Tween 20, pH 7.6) for 2 h at room temperature, the membranes were incubated with primary antibodies at 4°C overnight. The membranes were then washed 3 times in TBST for 10 min each time at room temperature, and then incubated with HRP-conjugated secondary antibodies. Finally, the membranes were visualized using DAB reagent (Dako Corporation, Denmark).

### shRNA transfection

β-catenin shRNAs (shRNA-1, shRNA-2) and the matching scrambled control sequences were purchased from GeneChemCo., Ltd (GeneChem, China) and used according to the manufacturer's protocol. β-catenin stably silenced cells were maintained in 5 μg/μL puromycin (Sigma-Aldrich, USA) and tested regularly by western analysis to ensure down-regulation.

### Immunofluorescence staining

Immunofluorescence staining was performed as previously described [18, 21]. The coverslips were incubated with rabbit anti-β-catenin primary antibodies at 4°C overnight and then followed by a FITC-conjugated anti-rabbit secondary antibody for 1 h at room temperature in the dark. After staining the nuclei with DAPI, the images were collected with a Nikon Eclipse Ti-S confocal microscope (Nikon, Japan).

### TOP/FOP Flash assay

HCT116 and HT29 cells were seeded in 24-well plates. After an incubation of 24 h/37°C, the cells were transfected with 200 ng luciferase reporter plasmid, TOP Flash (Merck Millipore, Germany), containing two sets of three copies of the TCF-binding site (wild type), or its control FOP Flash (Merck Millipore, Germany), containing mutated TCF-binding sites in combination with 2 ng of pRL-TK vector (Promega, USA) containing *Renilla* luciferase. Transfection was performed with Lipofectamine 2000 (Life Technologies, USA) and Opti-MEM reduced serum medium (Gibco, USA) according to the manufacturer's protocol. Cells were harvested 48 h post-transfection. Firefly and *Renilla* luciferase activities were measured by using the Dual Luciferase Reporter Assay (Promega, USA) according to the manufacturer's protocol. Firefly luciferase activity was normalized to *Renilla* luciferase activity and the transcript activity was shown as TOP fluorescent value/FOP fluorescent value.

### Animal models

All animal experiments were approved by the Ethics Committee of Ruijin Hospital (Shanghai, China). Male nude mice (Laboratory Animal Center of Shanghai, Academy of Science, Shanghai, China) aged 3–4 weeks were randomly divided into cages and injected subcutaneously with different numbers of HT29 cells (1 × 10^5^, 1 × 10^6^ and 1 × 10^7^ cells per mouse; *n* = 5–15 mice/group). Tumor growth was assessed by measuring the long and short diameters of the tumors every 5 days using calipers. Tumor volumes were calculated using the following formula: *V* = (length × width^2^)/2. Then 30 days post-inoculation, all the mice were sacrificed and the tumor xenografts were excised, weighed, fixed and embedded in paraffin.

### Clinical colorectal specimens and immunohistochemistry

This study was approved by the Ethics Committee of Ruijin Hospital (Shanghai, China), and all patients were fully informed of the experimental procedures. A total of 116 cases of colorectal specimens were collected from 2006–2008 and used to generate a CRC tissue microarray. Continuous sections (5 μm thick) were cut from the paraffin-embedded tissue microarray. After de-paraffinization and hydration, sections were incubated with endogenous peroxidase blocking solution and normal non-immune serum. After blocking, the continuous sections were incubated overnight at 4°C with respectively goat primary anti-NDRG1 polyclonal antibody (diluted 1:75), anti-β-catenin rabbit polyclonal antibody (1:50) and anti-CD44 mouse polyclonal antibody (1:100). The sections were then incubated with horseradish peroxidase (HRP)-conjugated second antibody for 1 h at room temperature. Finally, the slides were visualized with DAB and counterstained with hematoxylin. The staining intensity (SI) was evaluated as follows: 0–1 (negative): no staining or slightly darker than the background; 2 (moderate positive): obviously darker than the background, and 3 (strong positive): deep-colored staining. The percentage of positive cells (PP) was classified from a score of 0 to 4. The final immunoreactive score (IRS) was calculated as: IRS = SI × PP and IRS > 4 was considered positive expression.

### Statistical analysis

Statistical differences between experiment groups and corresponding control groups in the number of cells, colonies, or spheres were analyzed using Student's *t*-test. The two-way ANOVA was adopted in the analysis of inhibition rate caused by different concentrations of 5-FU. The correlations between NDRG1, β-catenin and CD44 expression in CRC were estimated by rank correlation and Spearman coefficients (r_s_) were calculated. All tests were analyzed with SPSS Statistics version 16.0 (IBM, USA) and a *p* < 0.05 was considered statistically significant.

## SUPPLEMENTARY TABLES



## Abbreviations

CRC: colorectal cancer
CSC: cancer stem cell-like cells
EMT: epithelial–mesenchymal transition
5-FU: 5-fluorouracil
NDRG1: N-myc downstream regulated gene-1
RT-PCR: reverse transcription-polymerase chain reaction.
