# Supplementary material for: The metastasis suppressor, NDRG1, inhibits “stemness” of colorectal cancer via down-regulation of nuclear β-catenin and CD44
Source: Oncotarget. 2015 Sep 18;6(32):33893–911. doi: 10.18632/oncotarget.5294 (PMC4741810; doi:10.18632/oncotarget.5294)
Supplement: Supplementary file 1 [file oncotarget-06-33893-s001.pdf]

SUPPLEMENTARY TABLES

Supplementary Table S1: The difference of NDRG1 expression in CRC tissues and adjacent normal tissues from 116 patient cases using immunohistochemical assessment

	Cases	NDRG1(+)	NDRG1(–)	*P* value
CRC tissue	116	39	77	
Normal tissue	116	66	50	0.001

Supplementary Table S2: The correlation between NDRG1, nuclear β-catenin and CD44 expression in 116 colorectal cancer specimens as judged by immunohistochemistry

	NDRG1	Nuclear β-catenin		CD44	
		positive(IRS > 4)	negative(IRS ≤ 4)	positive(IRS > 4)	negative(IRS ≤ 4)
positive(IRS > 4)	39	16	23	15	24
negative(IRS ≤ 4)	77	70	7	61	16
Cases	116	86	30	76	40
P value	$P < 0.001 (r_s = -0.558)$			$P < 0.001 (r_s = -0.375)$	

Supplementary Table S3: The relationship between NDRG1 expression and clinical-pathological data from 116 colorectal cancer specimens

	NNDRG1 (IRS >4)	NDRG1 (IRS ≤4)	Cases	*P* value
Samples:	39	77	116	
Gender:				0.620
Male	24	44	68	
Female	15	33	48	
Age(years):				0.522
≤65	16	36	52	
>65	23	41	64	
Tumor location:				0.687
Right colon	9	19	28	
Transverse colon	2	5	7	
Left colon	6	4	10	
Sigmoid colon	4	20	24	
Rectum	18	29	47	
Tumor size(cm2):				0.346
≤12	20	33	53	
>12	19	44	63	
Extent of invasion:				0.022*
T1+T2	15	16	31	
T3+T4	24	61	85	
Lymphatic metastasis:				0.011*
N0	25	31	56	
N1	10	32	42	
N2	4	14	18	
Metastasis:				0.614
M0	36	69	105	
M1	3	8	11	

Supplementary Table S4: The correlation between NDRG1, β-catenin and CD44 in tumor invasion and lymphatic metastatic cases of CRC as judged by immunohistochemistry

	NDRG1(+)	NDRG1(–)	β-catenin(+)	β-catenin(–)	CD44(+)	CD44(–)	Cases
T1+T2	15	16	19	12	17	14	31
T3+T4	24	61	67	18	59	26	85
N0	25	31	40	16	36	20	56
N1	10	32	34	8	27	15	42
N2	4	14	12	6	13	5	18
